# CXCR2 expression and postoperative complications affect long-term survival in patients with esophageal cancer

**DOI:** 10.1186/s12957-015-0658-7

**Published:** 2015-08-01

**Authors:** Tomohiko Nishi, Hiroya Takeuchi, Sachiko Matsuda, Masaharu Ogura, Hirofumi Kawakubo, Kazumasa Fukuda, Rieko Nakamura, Tsunehiro Takahashi, Norihito Wada, Yoshiro Saikawa, Tai Omori, Yuko Kitagawa

**Affiliations:** Department of Surgery, Keio University School of Medicine, 35 Shinanomachi, Shinjuku-ku, Tokyo, 160-8582 Japan

**Keywords:** CXCR2 protein, Postoperative complication, Esophagectomy

## Abstract

**Background:**

Esophagectomy is one of the most invasive surgical treatments for digestive tract cancer, and the blood levels of inflammatory cytokines such as interleukin-1, interleukin-6, and interleukin-8 are increased for several hours after surgery or in patients experiencing postoperative complications. CXCR2, an interleukin-8 receptor, is reportedly expressed in several carcinomas, and interleukin-8 signaling promotes cancer cell proliferation. The impact of postoperative complications following esophagectomy on long-term survival is controversial. In this study, we demonstrate the significance of CXCR2 expression and validate the effects of CXCR2 expression and postoperative complications on long-term prognosis of esophageal squamous cell carcinoma using resected specimens.

**Methods:**

Eighty-two specimens were sectioned from archived, paraffin-embedded tumor tissues obtained from patients with esophageal squamous cell carcinoma who underwent esophagectomy and extended lymphadenectomy for complete resection of cancer in our institute between 1997 and 2002. Immunohistochemistry was performed using a polyclonal antibody to CXCR2, and the correlation of stainability with clinicopathological factors and long-term survival was examined.

**Results:**

CXCR2 was expressed in 33 of 82 (40.2 %) specimens. In the CXCR2-positive group, the recurrence-free survival and overall survival rates of patients who developed postoperative complications were both significantly lower than those for patients who did not develop any complications. In contrast, in the CXCR2-negative group, there was no significant difference in long-term prognosis between patients with and without complications. CXCR2 positivity combined with postoperative complications was an independent risk factor for subsequent tumor recurrence, showing the highest hazard ratio.

**Conclusions:**

Our results suggest that the patients with CXCR2-positive esophageal cancer who develop postoperative complications have a poor prognosis and should be carefully followed.

**Trial registration:**

This study was approved by Keio University School of Medicine Ethics Committee with a trial registration number of 2011-241.

## Background

Esophageal cancer is the eighth most common cancer worldwide, with an estimated 482,000 new cases (3.8 % of the total) diagnosed in 2008, and the sixth most common cause of cancer-related death, with 407,000 deaths (5.4 % of the total) in 2008 [1]. The prognosis of esophageal cancer is generally poor because of its biological aggressiveness and anatomical characteristics. According to the American Joint Committee on Cancer (AJCC), the postoperative 5-year survival rate is approximately 90 % for stage I esophageal cancer, and it decreases to 45 % for stage II, 20 % for stage III, and only 10 % for stage IV [2].

Esophagectomy with radical lymphadenectomy represents the standard treatment for patients with clinically resectable esophageal cancer [3]. Recently, it was reported that preoperative chemotherapy or preoperative chemoradiotherapy followed by surgery improves prognosis compared with surgery alone [4]; however, the overall survival (OS) rate remains unsatisfactory. Therefore, there remains an urgent need to identify novel promising markers that can be used to predict the survival outcome as well as a new groundbreaking therapy for esophageal cancer.

Radical esophagectomy is one of the most invasive surgical treatments for digestive tract cancer, and the blood levels of inflammatory cytokines such as interleukin (IL)-1, IL-6, and IL-8 are increased for several hours after surgery. In addition, compared with other surgeries, esophagectomy is associated with a higher rate of complications, such as postoperative pneumonia and anastomotic leakage [5, 6]. Such postoperative complications are known to cause a cytokine storm [7].

Chemokines are small chemotactic cytokines that mediate communication among different cell types [8]. CXCR2, an IL-8 receptor, is a member of the G-protein–coupled receptor superfamily and the receptor of Glu–Leu–Arg (ELRþ) CXC chemokines. CXCL1, CXCL2, CXCL3, CXCL5, and CXCL7 bind specifically to CXCR2, while CXCL6 and CXCL8 (IL-8) are shared ligands of CXCR1 and CXCR2 [9]. CXCR2 expression has been demonstrated in neutrophils, monocytes, eosinophils, mast cells, basophils, lymphocytes, epithelial cells, and endothelial cells. In addition, CXCR2 is expressed on several carcinomas, and IL-8 signaling promotes proliferation, survival, and invasion of cancer cells as well as angiogenesis within tumors [9–12]. Previously, we reported that the expression of both IL-8 and CXCR2 in esophageal squamous cell carcinoma (ESCC) was an independent predictor of prognosis [13].

Some reports on a variety of malignancies demonstrated that postoperative complications may have either a positive or negative impact on long-term oncological survival [14–18]. The same thing goes for esophageal cancer. Some studies have shown that postoperative complications are negative predictors of the long-term survival after esophagectomy [19–21]. However, other studies reported that postoperative complications are not associated with long-term survival after esophagectomy [22–24].

It has been reported that serum and plasma levels of IL-8 are enhanced in patients with sepsis, acute lung injury (ALI), and pneumonia [25–27].

In our present study, we hypothesized that CXCR2 expression could stratify prognosis in ESCC patients with postoperative complications. To clarify the prognostic significance of CXCR2 expression and its relationship with common clinicopathological factors, particularly postoperative complications, in ESCC, we retrospectively examined 82 primary thoracic esophageal carcinomas using immunohistochemical staining.

## Methods

### Patients

Eighty-two patients who underwent transthoracic esophagectomy for ESCC at Keio University Hospital (Tokyo, Japan) between 1997 and August 2002 were examined. Patients were included in the study if they meet the following criteria: (1) histologically proven squamous cell carcinoma of the thoracic esophagus, (2) no microscopic residual tumor (R0), (3) no past history of neoadjuvant chemotherapy or chemoradiotherapy for ESCC, (4) no other cancer, and (5) no steroid use during the perioperative period. Informed consent was obtained from each patient enrolled in this study. All patients underwent two- or three-field lymph node dissection through right thoracotomy, either video-assisted thoracic surgery or open thoracotomy [2, 28]. Physical examinations before surgery did not detect high-risk factors for transthoracic esophagectomy under general anesthesia in any of the patients. Informed consent was obtained from each patient enrolled in this study. The study was approved by Keio University School of Medicine Ethics Committee.

### Morbidity following esophagectomy

The incidence of postoperative complications was determined by inspection of patients’ medical records. Vocal cord palsy was defined as hoarseness at the time of discharge. Pneumonia was defined as an abnormal shadow on a chest radiograph with fever (>38 °C), positive sputum, and/or a white blood cell count of ≥12,000/mm^3^. Postoperative complications were defined as complications of a grade equal to or higher than grade II according to the Clavien–Dindo classification [29]: grade I, any deviation from the normal postoperative course without the need for pharmacologic treatment or surgical, endoscopic, or radiologic intervention; grade II, requiring pharmacologic treatment with drugs; grade III, requiring surgical, endoscopic, or radiologic intervention; grade IV, life-threatening complication requiring intensive care unit management; and grade V, death.

### Follow-up

All patients were surveyed after esophagectomy in order to detect recurrences. Physical examinations and serum CEA and squamous cell carcinoma antigen tests were performed every 3 months. CT scans from the neck to the upper abdomen and upper gastrointestinal endoscopy were performed from every 6 months.

Tumor recurrence was classified as locoregional or distant. Locoregional recurrence was defined as recurrence at the primary site including the anastomosis and regional lymph nodes. It was described as distant recurrence when located in the liver, lungs, or other distant organs. OS was measured from the date of surgery to the date of death, or last follow-up. Recurrence-free survival (RFS) was measured from the date of surgery to the date of first evidence of relapse. For patients who had not relapsed or died, RFS was censored at the last date at which the absence of relapse was confirmed.

### Resected specimens and immunohistochemistry

For this study, tissue samples were obtained from esophageal specimens following surgical resection. For immunohistochemistry (IHC) analyses, slides were deparaffinized with xylene and rehydrated in a graded ethanol series staining. Antigen retrieval was performed using an enzyme solution (Proteinase K DAKO®, Glostrup, Denmark). Endogenous peroxidase activity was quenched with 0.5 % periodic acid for 10 min at room temperature. CXCR2 rabbit anti-human polyclonal antibodyLS-A803 (diluted at 1:250, LifeSpan BioSciences®, Seattle, WA, USA) was used as a primary antibody. Prepared sections were incubated overnight at 4 °C. Slides were subsequently incubated with labeled secondary antibody for 30 min at room temperature. We used biotinylated goat anti-rabbit IgG antibody BA-1000 (Vector®, CA, USA) as the secondary antibody. Peroxidase activity was detected with the enzyme substrate 3,3-diaminobenzidine tetrachloride solution. On the final section, the slide was lightly counterstained with hematoxylin and mounted. For each test case, a negative control was also included, which received the same treatment as the test slide, except that the primary antibody was omitted. We also used esophageal squamous epithelial cells without inflammation that lacked CXCR2 expression as negative controls. Positive controls (neutrophils and monocytes in the spleen for CXCR2 antibody, according to Emadi et al. [30]) were obtained to optimize the conditions. We evaluated CXCR2 expression in viable ESCC cells on slides that contained the most invasive tumor lesion. CXCR2 expression was assessed by two investigators (T. N. and H. T.) with no knowledge of the clinicopathological factors. Cases were considered as CXCR2-positive if more than 10 % of cancer cells exhibited cell wall staining.

We previously confirmed that CXCR2 protein is overexpressed in an ESCC cell line, TE4, by Western blot analysis. We also confirmed CXCR2 mRNA expression in TE4 using reverse transcriptase polymerase chain reaction [13].

### Statistical analysis

All data are presented as medians. Statistical significance was determined by a non-parametric Mann–Whitney *U* test or Fisher’s exact test. Cumulative survival rates for patient groups were calculated using the Kaplan–Meier method and were compared using the Mantel–Cox log-rank test. Cox proportional hazard models were used for multivariate analysis of variables for predicting postoperative survival. A *p* value of <0.05 was considered statistically significant. Statistical analyses were performed with SPSS v18.0 software (SPSS, Chicago, IL, USA).

## Results

### Patient demographics

The median age of patients was 59 years (range, 44–81 years) with a male/female ratio of 73/9 (Table 1). The pathologic stage, as determined using the sixth edition of the TNM/UICC classification, was I in 24 (29.3 %) patients, IIA in 7 (8.5 %) patients, IIB in 24 (29.3 %) patients, and III in 27 (32.9 %) patients (Table 2).

**Table 1:** Associations between CXCR2 expression and clinical background factors

Factors	All patients	CXCR2-positive	CXCR2-negative	*p* value
	(*n* = 82)	(*n* = 33)	(*n* = 49)	
Age (years)				0.81
≦49	3 (3.7 %)	1 (3.0 %)	2 (4.1 %)	
50–59	40 (48.8 %)	17 (51.5 %)	23 (46.9 %)	
60–69	31 (37.8 %)	13 (39.4 %)	18 (36.7 %)	
≧70	8 (9.8 %)	2 (6.1 %)	6 (12.2 %)	
Sex				0.33
Male	73 (89.0 %)	28 (84.8 %)	45 (91.8 %)	
Female	9 (11.0 %)	5 (15.2 %)	4 (8.2 %)	
Location				0.60
Upper	7 (8.5 %)	2 (6.1 %)	5 (10.2 %)	
Middle	52 (63.4 %)	20 (60.6 %)	32 (65.3 %)	
Lower	23 (28.0 %)	11 (33.3 %)	12 (24.5 %)	
Approach				0.82
Thoracotomy	61 (74.4 %)	25 (75.8 %)	36 (73.5 %)	
Thoracoscopy	21 (25.6 %)	8 (24.2 %)	13 (26.5 %)	
Adjuvant therapy				0.58
None	71 (86.6 %)	27 (81.8 %)	44 (89.8 %)	
Chemotherapy	9 (11.0 %)	5 (15.2 %)	4 (8.2 %)	
Chemoradiotherapy	2 (2.4 %)	1 (3.0 %)	1 (2.0 %)

**Table 2:** Associations between CXCR2 expression and pathological background factors

		All patients	CXCR2-positive	CXCR2-negative	*p* value
		(*n* = 82)	(*n* = 33)	(*n* = 49)	
pT factor	1	40 (48.8 %)	12 (36.4 %)	28 (57.1 %)	0.06
	2	10 (12.2 %)	7 (21.2 %)	3 (6.1 %)	
	3	32 (39.0 %)	14 (42.4 %)	18 (36.7 %)	
pN factor	0	31 (37.8 %)	12 (36.4 %)	19 (38.8 %)	0.83
	1	51 (62.2 %)	21 (63.6 %)	30 (61.2 %)	
Histological stage	I	24 (29.3 %)	9 (27.3 %)	15 (30.6 %)	0.95
	IIA	7 (8.5 %)	3 (9.1 %)	4 (8.2 %)	
	IIB	24 (29.3 %)	9 (27.3 %)	15 (30.6 %)	
	III	27 (32.9 %)	12 (36.4 %)	15 (30.6 %)	
Histological type	Well	17 (20.7 %)	7 (21.2 %)	10 (20.4 %)	0.65
	Moderately	57 (69.5 %)	24 (72.7 %)	33 (67.3 %)	
	Poorly	8 (9.8 %)	2 (6.1 %)	6 (12.2 %)	
Infiltrative growth pattern	INF-a	6 (7.3 %)	3 (9.1 %)	3 (6.1 %)	0.86
	INF-b	69 (84.1 %)	27 (81.8 %)	42 (85.7 %)	
	INF-c	7 (8.5 %)	3 (9.1 %)	4 (8.2 %)	
Lymphatic invasion	(+)	65 (79.3 %)	27 (81.8 %)	38 (77.6 %)	0.64
	(−)	17 (20.7 %)	6 (18.2 %)	11 (22.4 %)	
Venous invasion	(+)	39 (47.6 %)	18 (54.5 %)	21 (42.9 %)	0.30
	(−)	43 (52.4 %)	15 (45.5 %)	28 (57.1 %)	

*INF-a*, expansive growth of tumor nests with a well-demarcated border from surrounding tissue; *INF-b*, intermediate growth pattern, between INF-a and c; *INF-c*, infiltrative growth of tumor nests with an ill-defined border from surrounding tissue

**Fig. 1:**
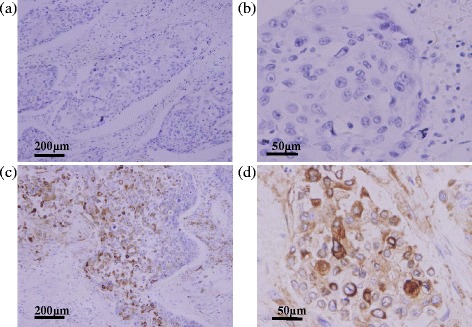
Immunohistochemistry of CXCR2. **a**, **b** Esophageal cancer cells are negative for CXCR2 expression. **c**, **d** CXCR2-positive staining is observed on the surface and in the cytoplasm of esophageal cancer cells. *Scale bars*, 200 and 50 μm

According to the results of IHC, CXCR2 expression was observed mainly in the cell wall of the cancer cells (Fig. 1), and 33 of the 82 cases (40.2 %) were evaluated as CXCR2-positive.

The patients were divided into two groups: a CXCR2-positive group and a CXCR2-negative group (Tables 1 and 2). There were no significant differences in clinicopathological background factors between the two groups according to the results of *χ*^2^ analysis. Of the 82 patients, 40 (48.8 %) developed postoperative complications (Table 3). As shown in Table 3, there were no significant differences in the rate of complications between the CXCR2-positive group and the CXCR2-negative group.

**Table 3:** Frequency of postoperative complications

Complications	All patients	CXCR2-positive	CXCR2-negative	*p* value
	(*n* = 82)	(*n* = 33)	(*n* = 49)	
Bleeding	1 (1.2 %)	0 (0.0 %)	1 (2.0 %)	>0.99
Grade IV	1 (1.2 %)	0 (0.0 %)	1 (2.0 %)	
Recurrent nerve palsy	5 (6.1 %)	4 (12.1 %)	1 (2.0 %)	0.15
Grade II	4 (4.9 %)	3 (9.1 %)	1 (2.0 %)	
Grade III	1 (1.2 %)	1 (3.0 %)	0 (0.0 %)	
Anastomotic leakage	14 (17.1 %)	5 (15.2 %)	9 (18.3 %)	0.70
Grade II	5 (6.1 %)	2 (6.1 %)	3 (6.1 %)	
Grade III	9 (11.0 %)	3 (3.0 %)	6 (12.2 %)	
Pneumonia	13 (15.9 %)	2 (6.1 %)	11 (22.4 %)	0.06
Grade II	9 (11.0 %)	1 (3.0 %)	8 (16.3 %)	
Grade III	3 (3.7 %)	1 (3.0 %)	2 (4.1 %)	
Grade IV	1 (1.2 %)	0 (0.0 %)	1 (2.0 %)	
Pyothorax	5 (6.1 %)	2 (6.1 %)	3 (6.1 %)	>0.99
Grade II	3 (3.7 %)	0 (0.0 %)	3 (6.1 %)	
Grade III	2 (2.4 %)	2 (6.1 %)	0 (0.0 %)	
Intestinal obstruction	2 (2.4 %)	1 (3.0 %)	1 (2.0 %)	>0.99
Grade II	1 (1.2 %)	1 (3.0 %)	0 (0.0 %)	
Grade III	1 (1.2 %)	0 (0.0 %)	1 (2.0 %)	
Bacteremia	1 (1.2 %)	1 (3.0 %)	0 (0.0 %)	0.40
Grade IV	1 (1.2 %)	1 (3.0 %)	0 (0.0 %)	
Catheter infection	3 (3.7 %)	3 (9.1 %)	0 (0.0 %)	0.06
Grade II	2 (2.4 %)	2 (6.1 %)	0 (0.0 %)	
Grade IV	1 (1.2 %)	1 (3.0 %)	0 (0.0 %)	
Anastomotic stenosis	1 (1.2 %)	1 (3.0 %)	0 (0.0 %)	0.40
Grade III	1 (1.2 %)	1 (3.0 %)	0 (0.0 %)	
Glottic edema	2 (2.4 %)	2 (6.1 %)	0 (0.0 %)	0.16
Grade III	2 (2.4 %)	2 (6.1 %)	0 (0.0 %)	
Intraabdominal abscess	1 (1.2 %)	1 (3.0 %)	0 (0.0 %)	0.40
Grade III	1 (1.2 %)	1 (3.0 %)	0 (0.0 %)	
Thrombosis	1 (1.2 %)	1 (3.0 %)	0 (0.0 %)	0.40
Grade II	1 (1.2 %)	1 (3.0 %)	0 (0.0 %)	
Any complication	40 (48.8 %)	17 (51.5 %)	23 (46.9 %)	0.68

### Patient survival

**Fig. 2:**
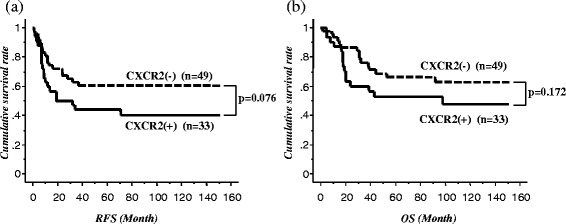
**a** Recurrence-free survival and **b** overall survival after esophagectomy in the two groups according to CXCR2 expression

The ESCC recurrence rate was 54.5 % (18/33 patients) in the CXCR2-positive group and 38.8 % (19/49 patients) in the CXCR2-negative group. Figure 2 shows the Kaplan–Meier curve for RFS and OS for these two groups. RFS of patients with CXCR2-positive tumors tended to be poorer than that of patients with CXCR2-negative tumors (*p* = 0.076; Fig. 2a). There was no significant difference in OS between the two groups (Fig. 2b).

**Fig. 3:**
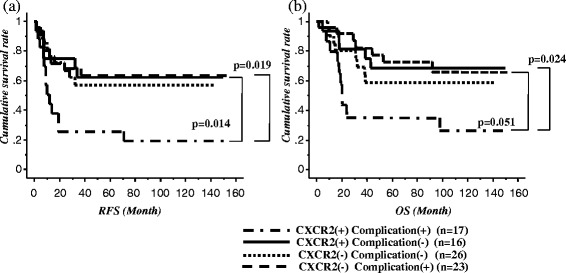
**a** Recurrence-free survival and **b** overall survival after esophagectomy among the four groups according to CXCR2 expression and postoperative complications

In the CXCR2-positive group, the RFS and OS rates of patients who developed postoperative complications were both significantly lower than those for patients who did not develop any complications (*p* = 0.019, Fig. 3a; *p* = 0.024, Fig. 3b). In contrast, in the CXCR2-negative group, there was no significant difference in RFS and OS between patients with and without complications (Fig. 3a, b, respectively).

Among patients who developed complications, RFS was significantly poorer in those with CXCR2-positive tumors than in those with CXCR2-negative tumors (*p* = 0.014; Fig. 3a). With regard to OS, the same tendency was observed (*p* = 0.051; Fig. 3b). Meanwhile, among patients who did not develop complications, there were no significant differences in RFS and OS between patients with CXCR2-positive tumors and those with CXCR2-negative tumors (Fig. 3a, b).

**Fig. 4:**
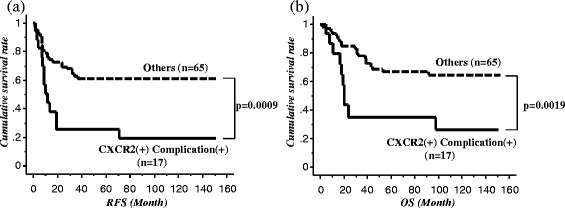
**a** Recurrence-free survival and **b** overall survival after esophagectomy between the CXCR2(+)/complication(+) group and the others

Therefore, we divided these 82 patients into two groups: those with CXCR2-positive tumors who developed postoperative complication(s) [CXCR2(+)/complication(+) group; *n* = 17 (20.7 %)] and all other patients [*n* = 65 (79.3 %)]. RFS for the CXCR2(+)/complication(+) group was significantly poorer than that for the other group (*p* = 0.0009; Fig. 4a). The same result was obtained for OS (*p* = 0.0019; Fig. 4b).

Univariate and multivariate analyses were performed to determine the predictors of subsequent tumor recurrence. We evaluated eight variables for survival prognosis by univariate analysis (Table 4). A depth of tumor invasion of pT2 or greater, lymph node metastasis, a disease stage of pStage IIA or greater, lymphatic invasion, venous invasion, and CXCR2(+)/complication(+) status were identified as predictive markers of RFS. We then performed multivariate analysis using Cox’s proportional hazard model for these six factors. The CXCR2(+)/complication(+) status was an independent risk factor for subsequent tumor recurrence, showing the highest hazard ratio compared with the other preoperative variables and each pathologic feature.

**Table 4:** Multivariate analysis of variables predicting recurrence-free survival with Cox’s proportional hazard model

Factors	Univariate *p* value	Multivariate	
		Hazard ratio (95 % CI)	*p* value
pT (pT1 or ≥pT2)	0.050	1.09 (0.44–2.69)	0.859
pN (pN0 or pN1)	0.015	1.04 (0.28–3.83)	0.952
pStage (pStageI vs other)	0.021	1.62 (0.29–8.93)	0.580
Lymphatic invasion (+) or (−)	0.025	1.27 (0.32–4.98)	0.740
Venous invasion (+) or (−)	0.009	1.62 (0.74–3.55)	0.230
Histological type (poor or others)	0.770		
Infiltrative growth pattern (INF-c or other)	0.067		
CXCR2(+)/complication(+) or other	0.001	2.51 (1.18–5.35)	0.017

*CI* confidence interval

### Recurrence pattern

Patients who relapsed with CXCR2-positive tumors had a significantly higher incidence of distant metastases (*p* = 0.0052; Table 5). Meanwhile, locoregional recurrence was not significantly correlated with CXCR2 expression.

**Table 5:** Relation between CXCR2 expression and sites of recurrence

	CXCR2-positive	CXCR2-negative	Total	*p* value
	(*n* = 33)	(*n* = 49)	(*n* = 82)	
Relapsing patients	19 (57.6 %)	18 (36.7 %)	37 (45.1 %)	0.063
Locoregional recurrence (+)	13 (39.4 %)	16 (32.7 %)	29 (35.4 %)	0.130
Locoregional recurrence (−)	6 (18.2 %)	2 (4.1 %)	8 (9.8 %)	
Distant recurrence (+)	14 (42.4 %)	5 (10.2 %)	19 (23.2 %)	0.005
Distant recurrence (−)	5 (15.2 %)	13 (26.5 %)	18 (22.0 %)	

## Discussion

The impact of postoperative complications following esophageal cancer surgery on long-term survival is controversial. Some studies report that patients with postoperative complications have a poor prognosis compared to patients with no complications after esophagectomy [19–21]. Meanwhile, other studies reported that postoperative complication is not associated with long-term survival after esophagectomy [22–24].

Many cancers have a complex chemokine network that influences tumor cell growth, survival, and migration as well as metastasis and angiogenesis [8]. These findings are consistent with the hypothesis that the systemic inflammatory response plays a significant role in stimulating tumor growth.

IL-8, one of the proinflammatory chemokines, is produced by macrophages and other cell types, including epithelial cells and endothelial cells. IL-8 is one of the major mediators of the inflammatory response. Its primary function is the induction of chemotaxis in its target cells, e.g., neutrophil granulocytes. IL-8 serves as a chemical signal that attracts neutrophils to the site of inflammation; therefore, it is also known as a neutrophil chemotactic factor [25].

It has been reported that serum and plasma levels of IL-8 are enhanced in patients with sepsis, ALI, and pneumonia [25–27]. Several studies also reported that serum IL-8 levels increase after an invasive procedure such as thoracoabdominal esophagectomy [31].

In the present study, we found that 40.2 % of ESCC specimens stained positively for CXCR2 expression in IHC, similar to the finding in our previous report [13].

Our results indicated that patients with CXCR2-positive tumors who developed postoperative complications were predicted to be at the greatest risk of subsequent tumor recurrence. In the present study, ESCC patients with CXCR2-positive tumors who developed postoperative complications had a significantly poorer RFS compared with other patients. Multivariate analysis demonstrated that the combination of CXCR2 expression and postoperative complications was an independent predictor of RFS. To the best of our knowledge, this is the first report to demonstrate that the combination of CXCR2 expression and postoperative complications affects long-term survival in patients with esophageal cancer.

The current study indicates that there is no relationship between CXCR2 expression and pathological background such as the depth of tumor invasion, lymph node metastasis, histological stages, lymphatic invasion, venous invasion, and histological type. Moreover, for patients who did not develop complications, there were no significant differences in RFS and OS between the CXCR2-positive group and CXCR2-negative group. These results suggest that CXCR2 expression alone is not a risk factor for ESCC prognosis and that CXCR2 expression accompanied by IL-8 exposure is a marker of poor prognosis.

Our study also demonstrated that CXCR2 expression in esophageal cancer was significantly correlated with the site of recurrence. Patients with CXCR2-positive tumors showed a significant tendency to develop distant metastases, and this result is consistent with that of several studies reporting that an IL-8 and CXCR2 network promotes tumor invasion, migration, and metastasis.

The present study has several limitations. First, because serum IL-8 levels in study patients were not measured, it remains unclear whether serum IL-8 levels in patients who developed postoperative complications were elevated. Second, the current study is a retrospective study; therefore, there were various sources of bias. A prospective study including the measurement of serum IL-8 levels is required in the future.

However, it is clear that the combination of CXCR2 expression and postoperative complications is the strongest risk factor for a poor prognosis in ESCC patients. Therefore, avoidance of postoperative complications and strict perioperative care are important for patients with CXCR2-positive tumors. Less toxic neoadjuvant treatments which cause less postoperative complications might be recommended in the case with CXCR2-positive tumors proven by preoperative biopsies. Furthermore, if a patient with CXCR2-positive tumors develops complications, a strict follow-up schedule should be followed and additional postoperative chemotherapy may be necessary.

Unfortunately, it is not clear whether the most appropriate treatment could be chosen by CXCR2 expression in this study. Further studies will be needed on how to choose the appropriate treatments for patients with CXCR2-positive tumors.

We previously reported that perioperative steroids reduce the inflammatory cytokines including IL-8 in an animal model [32]. Furthermore, several clinical studies have found that preoperative steroid administration reduces the production of inflammatory mediators [33, 34]. Administration of a steroid during the perioperative period and a CXCR2 antagonist may be effective for inhibiting inflammatory mediators in patients with CXCR2-positive tumors.

## Conclusions

In conclusion, we demonstrated that the patients with CXCR2-positive esophageal cancer who develop postoperative complications have a poor prognosis and should be carefully followed.

## References

[1] Ferlay J, Shin HR, Bray F, Forman D, Mathers C, Parkin DM (2010). Estimates of worldwide burden of cancer in 2008: GLOBOCAN 2008. Int J Cancer..

[2] Ando N, Ozawa S, Kitagawa Y, Shinozawa Y, Kitajima M (2000). Improvement in the results of surgical treatment of advanced squamous esophageal carcinoma during 15 consecutive years. Ann Surg..

[3] Lehnert T (1999). Multimodal therapy for squamous carcinoma of the oesophagus. Br J Surg..

[4] Ando N, Kato H, Igaki H, Shinoda M, Ozawa S, Shimizu H (2012). A randomized trial comparing postoperative adjuvant chemotherapy with cisplatin and 5-fluorouracil versus preoperative chemotherapy for localized advanced squamous cell carcinoma of the thoracic esophagus (JCOG9907). Ann Surg Oncol..

[5] Akamoto S, Okano K, Sano T, Yachida S, Izuishi K, Usuki H (2007). Neutrophil elastase inhibitor (Sivelestat) preserves antitumor immunity and reduces the inflammatory mediators associated with major surgery. Surg Today..

[6] Suda K, Kitagawa Y, Ozawa S, Saikawa Y, Ueda M, Ebina M (2006). Anti-high-mobility group box chromosomal protein 1 antibodies improve survival of rats with sepsis. World J Surg..

[7] Hirai T, Matsumoto H (2014). Regulating surgical oncotaxis to improve the outcomes in cancer patients. Surg Today..

[8] Balkwill F (2004). Cancer and the chemokine network. Nat Rev Cancer..

[9] Saintigny P, Mssarelli E, Lin S, Ahn YH, Chen Y, Goswarni S (2013). CXCR2 expression in tumor cells is a poor prognostic factor and promotes invasion and metastasis in lung adenocarcinoma. Cancer Res..

[10] Matsuo Y, Raimondo M, Woodward TA, Wallace MB, Gill KR, Tong Z (2009). CXC-chemokine/CXCR2 biological axis promotes angiogenesis in vitro and in vivo in pancreatic cancer. Int J Cancer..

[11] Maxwell PJ, Gallagher R, Seaton A, Wilson C, Scullin P, Pettigrew J (2007). HIF-1 and NF-kappaB-mediated upregulation of CXCR1 and CXCR2 expression promotes cell survival in hypoxic prostate cancer cells. Oncogene..

[12] Singh S, Nannuru KC, Sadanandam A, Varney ML, Singh RK (2009). CXCR1 and CXCR2 enhances human melanoma tumourigenesis, growth and invasion. Br J Cancer..

[13] Ogura M, Takeuchi H, Kawakubo H, Nishi T, Fukuda K, Nakamura R (2013). Clinical significance of CXCL-8/CXCR-2 network in esophageal squamous cell carcinoma. Surgery..

[14] Mcardle CS, Mcmillan DC, Hole DJ (2005). Impact of anastomotic leakage on long-term survival of patients undergoing curative resection for colorectal cancer. Br J Surg..

[15] Walker KG, Bell SW, Rickard MJ, Mehanna D, Dent OF, Chapuis PH (2004). Anastomotic leakage is predictive of diminished survival after potentially curative resection for colorectal cancer. Ann Surg..

[16] Kim DY, Roh JL, Choi JW, Choi SH, Nam SY, Kim SY (2014). Risk factors and survival outcomes for patients with anastomotic leakage after surgery for head and neck squamous cell carcinoma. Clin Exp Otorhinolaryngol..

[17] Jörgren F, Johansson R, Damber L, Lindmark G (2011). Anastomotic leakage after surgery for rectal cancer: a risk factor for local recurrence, distant metastasis and reduced cancer-specific survival?. Colorectal Dis..

[18] Branagan G, Finnis D (2005). Prognosis after anastomotic leakage in colorectal surgery. Dis Colon Rectum..

[19] Rizk NP, Bach PB, Schrag D, Bains MS, Turnbull AD, Karpeh M (2004). The impact of complications on outcomes after resection for esophageal and gastroesophageal junction carcinoma. J Am Coll Surg..

[20] Lerut T, Moons J, Coosemans W, Van Raemdonck D, De Leyn P, Decaluwé H (2009). Postoperative complications after transthoracic esophagectomy for cancer of the esophagus and gastroesophageal junction are correlated with early cancer recurrence: role of systematic grading of complications using the modified Clavien classification. Ann Surg.

[21] Takeuchi H, Saikawa Y, Oyama T, Ozawa S, Suda K, Wada N (2010). Factors influencing the long-term survival in patients with esophageal cancer who underwent esophagectomy after chemoradiotherapy. World J Surg.

[22] Ferri LE, Law S, Wong KH, Kwok KF, Wong J (2006). The influence of technical complications on postoperative outcome and survival after esophagectomy. Ann Surg Oncol.

[23] Ancona E, Cagol M, Epifani M, Cavallin F, Zaninotto G, Castoro C (2006). Surgical complications do not affect longterm survival after esophagectomy for carcinoma of the thoracic esophagus and cardia. J Am Coll Surg.

[24] Xia BT, Rosato EL, Chojnacki KA, Crawford AG, Weksler B, Berger AC (2013). Major perioperative morbidity does not affect long-term survival in patients undergoing esophagectomy for cancer of the esophagus or gastroesophageal junction. World J Surg.

[25] Chaudhry H, Zhou J, Zhong Y, Ali MM, McGuire F, Nagarkatti PS (2013). Role of cytokines as a double-edged sword in sepsis. In Vivo.

[26] Paats MS, Bergen IM, Hanselaar WE, Groeninx van Zoelen EC, Hoogsteden HC, Hendriks RW (2013). Local and systemic cytokine profiles in nonsevere and severe community-acquired pneumonia. Eur Respir J.

[27] Chollet-Martin S, Montravers P, Gibert C, Elbim C, Desmonts JM, Fagon JY (1993). High levels of interleukin-8 in the blood and alveolar spaces of patients with pneumonia and adult respiratory distress syndrome. Infect Immun.

[28] Kaburagi T, Takeuchi H, Kawakubo H, Omori T, Ozawa S, Kitagawa Y (2014). Clinical utility of a novel hybrid position combining the left lateral decubitus and prone positions during thoracoscopic esophagectomy. World J Surg.

[29] Dindo D, Demartines N, Clavien PA (2004). Classification of surgical complications: a new proposal with evaluation in a cohort of 6336 patients and results of a survey. Ann Surg.

[30] Emadi S, Clay D, Desterke C, Guerton B, Maquarre E, Charpentier A (2005). IL-8 and its CXCR1 and CXCR2 receptors participate in the control of megakaryocytic proliferation, differentiation, and ploidy in myeloid metaplasia with myelofibrosis. Blood.

[31] Frick VO, Justinger C, Rubie C, Graeber S, Schilling MK, Lindemann W (2012). Thoracotomy procedures effect cytokine levels after thoracoabdominal esophagectomy. Oncol Rep.

[32] Nakamura E, Kitagawa Y, Ozawa S, Suda K, Ando N, Ueda M (2006). Role of steroid administration to reduce inflammation after thoracotomy in a rat surgical stress model. J Surg Res.

[33] Shimada H, Ochiai T, Okazumi S, Matsubara H, Nabeya Y, Miyazawa Y (2000). Clinical benefits of steroid therapy on surgical stress in patients with esophageal cancer. Surgery.

[34] Raimondi AM, Guimarães HP, Amaral JL, Leal PH (2006). Perioperative glucocorticoid administration for prevention of systemic organ failure in patients undergoing esophageal resection for esophageal carcinoma. Sao Paulo Med J.

